# Further Observations on the Internal Use of the Hydrocyanic (Prussic) Acid, in Pulmonary Complaints; Chronic Catarrhs; Spasmodic Coughs; Asthma; Hooping-Cough; and Some Other Diseases. With Full Directions for the Preparation and Administration of That Medicine

**Published:** 1819-10-01

**Authors:** 


*Further Observations on the Internal Use of the Hydrocyanic (Prussic) Acid, in Pulmonary Complaints; Chronic Catarrhs; Spasmodic Coughs; Asthma; Hooping-Cough; and some other Diseases. With full Directions for the Preparation and Administration of that Medicine.* By A. B. Granville, M. D. F. R. S. F. L S. M. R. I. Physician in ordinary to His Royal Highness the Duke of Clarence; Licenciate of the Royal College of Physicians of London; and Physician-Accoucheur to the Westminster General Dispensary. London, 1819. Octavo, pp. 82.

Dr. Granville, after detailing the modes of preparing, and the physical properties of, the Prussic acid, prefaces his account of its effects upon the human frame by remarking, that though poisonous in itself, like many of our best remedies, it may be used with perfect safety and advantage when properly administered; and, that no case has yet been recorded in which it has proved either fatal or injurious.

Another objection which he admits to be better founded, viz. that it has failed in producing any good effect in some of the complaints in which it has been exhibited, the author thinks *only* goes to prove a want of sufficient knowledge in the person employing it; but, we would ask, may it not also, peradventure, prove the incompetency of the medicine employed? The following are the effects of this remedy as stated by Dr. G.

"The Prussic acid is evidently sedative, more so even than opium; but its specific mode of action is somewhat different, both as to its progress and effect, from that of the latter substance. The Prussic acid, when administered to a patient exhausted by disease, and by the means employed to cure it, appears to exert an immediate influence upon the nervous system; it gradually diminishes all irritability, checks a too rapid circulation, and calms many of the symptoms of fever. If a dry cough be present, it promotes expectoration in the first instance, and subsequently stops the cough itself. The spirits, before exalted, soon feel the quieting impression of the acid; they become subdued; the speech, countenance, even the expresion of the eyes, assume a character of unusual meekness; there is a relief from pain and actual suffering; the patient feels it, and is grateful: sleep comes on undisturbed, respiration is soft, and the pulse more quiet than at other periods of the complaint, having lost the thumping beat of irritation. In some few cases these sedative effects are so much more considerable, that the patient expresses that he feels himself as if only *half alive*. On those occasions there is an *apparent* entire prostration of strength, great lowness of spirits, and unwillingness to move, speak, or take food; life seems suspended, yet the head and mind remain clear and intelligent; there is a total absence of pain; neither does the patient complain of any symptom of local or general irritation; the heat of the skin is natural, and the pulse, in the midst of this dead suspense, continues its course steadily and quietly. This state of things lasts from twelve to twenty-four hours, when it ceases; and every organ is gradually restored to its former elasticity.

"But it should be borne in mind that such instances of great depression, produced by the acid, are extremely rare, and indeed seldom occur where tonics, or a proper quantity of nourishment can be given to the patient, at the same time he is taking the acid. There is scarcely a remedy which does not, more or less, present some anomalies in its effects, dependant on the particular idiosyncrasy of the patient. In some few cases the Prussic acid disagrees with the stomach, and then it neither can, nor ought to be, persisted in: it also occasions giddiness in some individuals, in which case it is necessary, either to discontinue it, or diminish the dose, and associate it with slight stimulants. Opium, henbane, &c. have often, in this respect, exhibited the same occasional deviations from their usual mode of action.

"But the Prussic acid has never yet been found to produce the head-ache, and heaviness occasioned by laudanum; the fluttering and palpitations brought on by hemlock; nor the parched mouth, and irritability of the throat, arising from the action of belladonna. The Prussic acid moreover, acts gently on the bowels, in the first instance, and when after some days they seem to fall into a torpid state, the mildest medicines, and those in smaller quantities than usual, suffice to produce the desired effect, when combined with the acid."

The author next proceeds to enumerate the diseases in which it has proved beneficial. Its effects "in almost every kind of cough, particularly of a spasmodic nature, are highly satisfactory." "In hectic fever it affords ease; lowers the pulse; diminishes the number of paroxysms; works a favourable change in the action of the lungs and their circulation, while the morbid heat of the skin, and the circular flushes of the cheeks, gradually disappear. The night sweats are also soon suspended." p. 21. "In the treatment of confirmed consumption, the prussic acid, even at the approach of death, is the most advantageous palliative that can be employed. Greatly superior, indeed, to any hitherto adopted." p. 22. Asthmatic complaints are also stated to have been greatly relieved by it; and from analogy, the author is sanguine enough to expect much benefit from it in spasms of the stomach and diaphragm, and even in "locked jaw, tic douloureux, and, perhaps, hydrophobia!" p. 23. "Credat Judæus appella." The following instance, however, we can fully credit: "but I have had no experience of its utility in such affections."

Dr. G. has successfully employed this medicine in the dry convulsive spasmodic cough, which may be called sympathetic, as depending entirely on a morbid state of the liver, stomach, spleen, &c. With regard to hooping cough, "it may be stated, without presumption, that no case need be suffered to proceed longer than eight or ten days if the prussic acid be timely and cautiously administered; and, it is singular that children bear the action of this sedative medicine, in small doses, better than adults," p. 24. In inflammatory affections of the lungs, which have a tendency to recur on the slightest cause, and to terminate in hectic and purulent expectoration, and after depletion cannot be carried any further, Dr. G. thinks the prussic acid will produce the most advantageous effects; and also in those pains which attend and follow abortions, and in hæmoptysis. p. 25, 6, 7. Some cases follow, illustrative of its good effects in consumptive and other cases above mentioned, by the Author, Dr. Majendie, Mangini, Scudamore, and Mr. Thompson, of Sloane Street; and, according to their observations, no bad effects had ever resulted from this medicine, though great care is admitted to be requisite in its exhibition.

Towards the conclusion of the volume is a valuable communication from the latter gentleman, with a detail of four cases, in which he exhibited this medicine. He observes, "I have prescribed the acid in a great number of instances, with variable success; but the benefit it has produced is amply sufficient to authorize me to add my testimony in favour of its value as an important addition to the host of direct sedatives." p. 67.

The 8th Section is occupied with the mode of prescribing the prussic, or hydro-cyanic acid. It decomposes most of the salts used in medicine, particularly those of antimony and soda. Its affinity for the alkalies and earths is inconsiderable; that for the former is so weak, that even carbonic acid displaces it. Hence it may be given with carbonate of potash, forming one of the most successful modes of prescribing it in spasmodic and hooping coughs. It cannot be administered with the sulphurets. It may be given in vegetable infusions, with the addition of syrups if necessary. With tonics it may be associated with real advantage, as with the filtered infusions of bark, columbo, cascarilla, or even sarsaparilla, or in incipient pulmonary complaints in a strong decoction of lichen islandicus. It is indispensably necessary to use no other than distilled water in all prescriptions with the prussic acid; otherwise decomposition will take place. p. 60, 63. Some formulæ follow, in which eight or ten minims of the acid are exhibited in six or eight ounces of the vehicle; a table spoon full every two or three hours. p. 63. When the prussic acid produces nausea, vomiting, or dizziness, which it will do in some individuals on the very first or second day, it is advisable to abandon it immediately, for there is no chance of its ever agreeing with the patient; but such effects have not occurred above five or six times in 100 cases in which it has been exhibited. p. 39. During the first days it proves gently aperient; and when it has this effect, the mildest purgatives will suffice to produce the necessary evacuation. Mr. Thompson states his general practice in catarrhal affections and chronic coughs to have been, after purging, to give two minims of the acid in a spoonful of distilled water or almond emulsion, every two or three hours during the day, increasing the quantity two or three minims on the whole portion taken in the twenty-four hours, every day, until the cough was subdued. p. 70. The greatest amount to which it has been carried by him was twenty-four minims in the day to an adult, and six to an infant. For infants between four months and a year old, he prescribed two minims in ℥ix of distilled water, with ℥j of the syrup of Tolu, coch. min. ji. 3tiis. horis sumenda. As to its modus operandi, he observes, "the prussic acid, when taken into the stomach, produces its action on the circulating system, evidently through the medium of the nerves, the energy of which it considerably lessens and even altogether destroys when the dose is sufficiently strong. In no case have I remarked that any excitement precedes its sedative effect, a circumstance which distinguishes it from every other substance belonging to the class of narcotics." p. 67.

There are some typographical mistakes which ought to have been noticed in a table of errata, as in the formula, page, 76, and at page 81, *ob*noxious is used instead of noxious. In presenting a short but faithful analysis of this little volume, we will not say, "cras credemus, hodie nihil," but we shall feel much contented, and deem the prussic acid no contemptible acquisition, if experience shall prove it to deserve even a moderate competency of the virtues and efficacy here ascribed to it. We recommend this little work to the perusal of our brethren.

